# Cisterna magna reconstruction with arachnoid suturing in brainstem surgery

**DOI:** 10.3171/2019.10.FocusVid.19455

**Published:** 2019-10-01

**Authors:** Abdullah Keleş, Mehmet Volkan Harput, Uğur Türe

**Affiliations:** Department of Neurosurgery, Yeditepe University School of Medicine, Istanbul, Turkey

**Keywords:** arachnoid suturing, brainstem surgery, brainstem hemangioblastoma, cisterna magna reconstruction, meningocerebral adhesion, posterior fossa reconstruction, video

## Abstract

We present an effective and easily applied technique for cisterna magna reconstruction with arachnoid suturing in brainstem surgery. Suturing with 10-0 monofilament was done in a patient with a medulla oblongata hemangioblastoma (diagnosed von Hippel-Lindau disease). Seven years later, follow-up imaging revealed a new lesion close to the previous one and the patient underwent reoperation. The craniotomy and dural incision were repeated, and the intact arachnoid was visualized with no meningocerebral adhesions. This technique preserves normal anatomic landmarks and facilitates and shortens dissection in reoperations, almost like a virgin case. We propose this technique for every lower brainstem and fourth ventricle procedure.

The video can be found here: https://youtu.be/RKMcSoK6ycY.

## Transcript

The art of microneurosurgery relies on atraumatic exploration, total removal of a lesion without harming normal structures, and, as much as possible, reconstruction of the normal anatomy.

Here, we present a simple, effective and easily applied technique to prevent dense meningocerebral adhesions. This technique preserves normal anatomic landmarks, facilitates and shortens dissection during reoperation.

In this video, we demonstrate cisterna magna reconstruction with arachnoid suturing for state-of-the-art brainstem surgery (Spetzler et al., 2019).

This is the case of a 28-year-old female previously diagnosed with von Hippel-Lindau disease. She came to our institution in January of 2008 with a monthlong history of left-sided hemihypesthesia, numbness, and dysphagia.

The patient’s preoperative MRI (magnetic resonance imaging) revealed a solid lesion on the left posterior medulla oblongata with a macrocystic component. The solid component showed contrast enhancement on T1-weighted postcontrast images. The MRI findings revealed a hemangioblastoma of the medulla oblongata.

With preoperative vertebral angiography, major feeding arteries and draining veins were determined. In this case, the lesion’s major feeding artery originated from the left PICA.

For surgery, the patient was placed prone and a median inferior suboccipital craniotomy was done.

The dura was opened in Y-shape fashion.

The arachnoid of the cisterna magna was cautiously opened in the midline with microscissors.

Opening with sharp dissection and microneurosurgical techniques is important for successful arachnoid closure.

This technique has been described by Professor Yaşargil, and we utilize it in every brainstem and fourth ventricle surgery whenever possible (Yaşargil, 1996).

After cutting the arachnoid trabeculae, the arachnoid was reflected laterally and fixed to the dura with hemoclips.

A second microlesion was noticed on the posterior midline surface of the medulla oblongata. This lesion was not seen on preoperative studies.

Before tumor dissection, this video angiography gave us detailed information on feeding arteries intraoperatively.

Tumor dissection started with coagulation of the feeding arteries one by one.

All vessels related to the lesion except the main draining vein were coagulated and cut.

Tumor dissection continued circumferentially along the cleavage plane. Transsection of the tumor must be avoided because of the difficulty in controlling its profuse bleeding.

The main draining vein was cut at the end of the dissection.

The lesion was removed in one piece after the last attachment was cut.

Frequent warm saline irrigation protects the arachnoid from drying out and helps maintain hemostasis during surgery.

When necessary, bipolar coagulation on a very low setting is enough for dissection and hemostasis.

The vessels related to the second lesion were coagulated and cut. This lesion was also removed in one piece.

After tumor removal, the integrity of the normal vasculature was visualized with ICG video angiography.

Then, the hemoclips were removed and the edges of the arachnoid were brought together.

The first stitch was placed in the middle of the arachnoid incision with 10-0 monofilament nylon sutures.

After the first knot, the cranial part of the incision was sutured with a continuous suturing technique.

Small arachnoid defects close to the edge of the arachnoid can be included in the suture line.

The caudal part of the incision was also closed with the same suturing technique.

After appropriate arachnoid suturing, the dura was closed with a dural graft in a watertight fashion.

The patient’s postoperative course was uneventful, and she was discharged on postoperative day 5.

Histopathological analysis revealed a hemangioblastoma of WHO grade I.

The 3-month follow-up MRI showed total removal of the tumor.

The patient was neurologically intact.

After 7 years, in January of 2015, a follow-up MRI revealed a new lesion located at the medulla oblongata. The patient was neurologically intact.

In this case, the main feeding artery originated from the left PICA.

The patient was placed prone, and we used the same skin incision and craniotomy.

During the dural opening, the cisterna magna seemed to be intact, almost as if it had been untouched.

This clean surgical area without meningocerebral adhesions is invaluable for reopening in such cases.

The arachnoid of the cisterna magna was carefully opened with sharp dissection at the midline. At this stage, dissection was much easier than anticipated because there were no adhesions between the arachnoid and medulla oblongata.

The location of the new lesion was more superior than that of the previous lesions, confirming that this was a new hemangioblastoma.

Before tumor dissection, the major feeding arteries and draining veins were identified with ICG video angiography.

Then we coagulated and cut each feeding artery one by one.

Because the lesion was well encapsulated, the tumor cleavage plane was easily recognized. With bipolar coagulation on a low-power setting, the tumor and normal tissue were separated along the cleavage plane.

The cystic component of the lesion was opened and its contents evacuated.

After detaching the tumor from the cerebellar tonsil, the main draining vein was coagulated and cut. The tumor was removed in one piece.

While exploring the surgical cavity after tumor removal, we noticed a very small new lesion. This lesion was not seen on preoperative studies. We coagulated and removed this lesion.

As in the previous surgery, we needed to keep in mind that there may be new lesions that were not recognized on preoperative studies.

Adequate hemostasis was achieved with low-power bipolar coagulation and warm saline irrigation.

ICG video angiography was repeated, and the integrity of normal vascularization and total removal were confirmed.

The arachnoid edges were brought as close as possible and partially reconstituted with 10-0 monofilament nylon sutures.

Previously, we have described the benefit of placing a thin layer of gelatin sponge subdurally during the closure. This practice prevents meningocerebral adhesions (Gonzalez-Lopez et al., 2015).

The dura was closed in watertight fashion with a dural graft.

The patient’s postoperative course was uneventful and she was neurologically intact.

She was discharged on postoperative day 4.

The histopathological diagnosis was the same as the previous report, a WHO grade I hemangioblastoma.

Early postoperative and 3-month follow-up MRI showed total excision of the lesion and no other lesions.

As we demonstrated in this video, reconstruction of the cisterna magna with arachnoid membrane suturing is an easy, effective technique to prevent postoperative meningocerebral adhesions.

We strongly recommend this technique for each brainstem and fourth ventricle surgery that uses a posterior midline approach.

## Time points

1:01 Case presentation and preoperative MRI (first surgery)
2:00 Patient positioning and craniotomy
2:15 Cisterna magna arachnoid dissection
3:11 Removal of the hemangioblastoma
4:39 Arachnoid suturing
5:36 3-month postoperative MR images
5:55 Case presentation and preoperative MRI (second surgery)
6:25 Cisterna magna arachnoid dissection
7:06 Removal of the hemangioblastoma
9:23 3-month postoperative MR images

## Acknowledgments

The authors thank Aylin Akçaoğlu for the medical illustration and Julie Yamamoto for editing the narration and the abstract.

## Disclosures

The authors report no conflict of interest concerning the materials or methods used in this study or the findings specified in this publication.
